# Drug Retention Rates and Treatment Discontinuation among Anti-TNF-*α* Agents in Psoriatic Arthritis and Ankylosing Spondylitis in Clinical Practice

**DOI:** 10.1155/2014/862969

**Published:** 2014-07-08

**Authors:** Marta Fabbroni, Luca Cantarini, Francesco Caso, Luisa Costa, Veronica Anna Pagano, Bruno Frediani, Stefania Manganelli, Mauro Galeazzi

**Affiliations:** ^1^Rheumatology Unit, Research Center of Systemic Autoimmune and Autoinflammatory Diseases, Policlinico Le Scotte, University of Siena, Viale Bracci 1, 53100 Siena, Italy; ^2^Rheumatology Unit, Department of Medicine DIMED, University of Padova, Via Giustiniani 2, 35128 Padova, Italy; ^3^Rheumatology Unit, Department of Clinical Medicine and Surgery, University Federico II, Via S. Pansini 5, 80131 Naples, Italy; ^4^TFS Develop, Viale Parioli 12, 00197 Rome, Italy

## Abstract

*Objective. *The study aim was to determine treatment persistence rates and to identify causes of discontinuation in psoriatic arthritis (PsA) and ankylosing spondylitis (AS) patients in clinical practice.* Methods.* Patients treated with adalimumab (ADA), etanercept (ETA), or infliximab (INF) were retrospectively included. Treatment persistence rates were analyzed by means of a stepwise logistic regression. Differences between therapy duration were assessed by means of an analysis of variance model (ANOVA), while a chi-square test was used to evaluate relationships between therapies and causes of treatment discontinuation and the administration of concomitant disease-modifying antirheumatic drugs (DMARDs) among therapies and types of disease considering completed courses of therapy versus courses that were discontinued.* Results.* 268 patients received a total of 353 anti-TNF treatment courses (97 ADA, 180 ETA, and 76 INF). Comparison among therapies showed significant difference regarding the treatment persistence rates due to the contrast between ETA and INF (*P* = 0.0062). We observed that 84.7% of patients were still responding after 6 months of follow-up. Comparison among diseases showed that there were significant differences between PsA and AS (*P* = 0.0073) and PsA and PsA with predominant axial involvement (*P* = 0.0467) in terms of duration of the therapy, while there were no significant differences with regard to the persistence rate.* Conclusions.* In this cohort, anti-TNF-*α* therapy was associated with high drug persistence rates. As in rheumatoid arthritis, switching to another anti-TNF-*α* agent can be an effective option when, during the treatment of AS or PsA, therapy is suspended because of inefficacy or an adverse event. Combination therapy with DMARDs was associated with a better persistence rate.

## 1. Introduction

Psoriatic arthritis (PsA) and ankylosing spondylitis (AS) belong to the group of inflammatory spondyloarthritis (SpA) [1], of which the former is characterized by specific association with skin and/or nail psoriasis [2, 3], and both can be associated with other possible systemic features [4–10].

SpA therapy has been revolutionized by increasing knowledge of the pathogenetic mechanisms of the disease, involving dysfunction and oversecretion of multiple proinflammatory molecules, in particular tumor necrosis factor- (TNF-) *α* [11–13].

Thus, in the last decade, the introduction of TNF-*α* blockers has opened new horizons for patients and rheumatologists in the treatment of SpA [12, 13].

Currently, among five biological agents used in SpA therapies, the first three FDA-approved ones are human anti-tumor necrosis factor-alpha monoclonal antibody, adalimumab (ADA) (40 mg subcutaneously biweekly), human soluble TNF receptor fusion protein, etanercept (ETA) (50 mg subcutaneously once weekly or 25 mg twice weekly), and chimeric mouse-human anti-TNF-*α* monoclonal antibody, infliximab (INF) (5 mg/kg intravenous infusion at weeks 0, 2, and 6 and bimonthly) [14]. These agents have been largely demonstrated to be effective at reducing disease activity and controlling joint damage and various aspects of the diseases and reasonably safe both in PsA and in AS [15–24].

However, in spite of its generally high efficacy, some patients with AS or PsA can be refractory to anti-TNF-*α* therapy, may lose responsiveness, or develop drug intolerance over time. As in other rheumatic conditions, such as rheumatoid arthritis (RA) [25], a switch to another TNF-*α* antagonist, due to ineffectiveness or occurrence of adverse events, can often restore therapeutic response [26–32].

The relatively recent use of these agents has underscored the importance of clarifying anti-TNF-*α* retention rates in the context of routine clinical practice.

Hence in this study we assessed, on the basis of retrospective data, the persistence of anti-TNF-*α* agents in a cohort of patients undergoing long-term treatment for spondyloarthritis in a real-life clinical setting.

## 2. Methods

We performed a retrospective and observational analysis of clinical charts of consecutive SpA Caucasian patients receiving at least one of the three TNF-*α* blockers (ADA, ETA and INF) at the Outpatient Rheumatology Clinic at the University of Siena, from May 2008 to March 2014. Being a retrospective observational study, only local ethical committee notification was required.

Psoriatic arthritis was classified on the basis of CASPAR criteria [33] and AS was classified on the basis of modified NY criteria [34].

Thus, as a part of routine clinical practice, we analyzed data on therapy with TNF-*α* blockers, administrated in accordance with specific scheduled drugs recommendations.

Effectiveness was determined on the basis of DAS28 scores [35] and EULAR criteria for psoriatic arthritis [36] and on the basis of the BASDAI [37] and BASFI [38–41] instruments for AS. Further treatment decisions were based on these outcomes. Patients whose anti-TNF-*α* treatment was not modified during follow-up visits were responders according to the EULAR criteria and DAS28 score for PsA, and in the case of Ax-SpA and AS showed an adequate BASDAI response.

Treatment persistence rates were analyzed by means of a stepwise logistic regression using the variables selected. The factors included in the analysis were type of therapy, type of disease, axial involvement, gender, and the previous and concomitant administration of disease-modifying antirheumatic drugs (DMARDs). Differences in therapy duration, based on type of disease, were assessed by an analysis of variance model (ANOVA) using the same factors considered in the logistic model. The same model was used to analyze two subgroups: persistent therapy courses and courses that were discontinued. Baseline characteristics were examined by means of parametric or nonparametric methods, as appropriate.

A chi-square test was used to evaluate relationships between the therapies and causes of treatment discontinuation (categorized as adverse events, drug inefficacy, remission, or lack of compliance) and the administration of concomitant DMARDs for each disease (AS and PsA) taking axial or peripheral involvement into consideration, as well as between persistent therapy courses and courses that were discontinued. The same test was used to investigate differences in drug persistence among the 3 anti-TNF agents in the largest group (PsA group). For all the models, a *P* value of <0.05 as significant has been considered.

## 3. Results

A total of 268 SpA patients (47% women), including 213 with PsA and 55 with AS, receiving treatment with an anti-TNF-*α* agent (ADA, ETA or INF) were included in the study. In the PsA group, 23 patients showed predominantly axial involvement fulfilling the ASAS criteria for the diagnosis of axial spondyloarthritis (Ax-SpA) [42, 43].

Demographic characteristics of the studied cohort are reported in Table 1.

Overall, 353 anti-TNF-*α* treatment courses were administered. Mean follow-up was 33.7 months for the cohort and varied according to the timing of the availability of each biologic therapy in the clinic (Table 1). To date, 212 patients have been tracked for at least 12 months. Regarding the duration of individual anti-TNF-*α* therapy courses, mean (±SD) duration was 14.6 (±14.2) months for subjects receiving ADA, 28.5 (±23.9) months for ETA and 33.3 (±25.9) months for INF (*P* value < 0.0001).

A withdrawal was required in 98 anti-TNF-*α* treatment courses: 28/97 with ADA (28.9%), 41/180 with ETA (22.8%) and 29/76 with INF (38.2%). Biologic therapy was suspended because of adverse events in 11.3% of subjects receiving ADA, in 11.7% receiving ETA and in 11% receiving INF. In particular, the major side effects were cutaneous or mucosal reactions (25%), infections (23%), hepatic, renal, or hematopoietic dysfunction (16%), cardiovascular disease (8%), neurological or psychiatric disorders (6%), neoplasm (7%) and other events (15%). Biologic therapy withdrawal due to inefficacy occurred in 15.5% of subjects receiving ADA, in 9.4% receiving ETA and in 26.3% receiving INF. In two patients receiving INF and one receiving ETA, anti-TNF-*α* therapy was suspended upon achievement of clinical remission. One patient was discontinued due to lack of compliance. There were no statistically significant differences among therapies regarding causes of discontinuation (*P* value = 0.0783).

**Figure 1:**
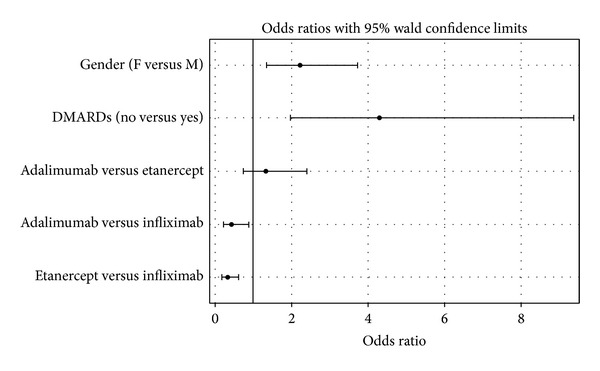
Odds ratios for treatment persistence rates.

Regarding anti-TNF-*α* treatment persistence rates, logistic model results showed a statistically significant difference between ETA and INF (Odds Ratio 0.329, CI (0.176, 0.615), *P* value = 0.0058), while the other two comparisons were not statistically significant (ADA versus ETA: Odds Ratio 1.330, CI (0.740, 2.392), *P* value = 0.3409; ADA versus INF: Odds Ratio 0.438, CI (0.218, 0.877), *P* value = 0.3458) (Figure 1). The administration of concomitant DMARDs (No versus Yes: Odds Ratio 4.294, CI (1.967–9.371)) and gender (Female versus Male: Odds Ratio 2.227, CI (1.335–3.716)) were also associated with drug persistence rates. Taking into consideration only the largest group of PsA patients, the biologic drug retention rates did not differ among the three anti-TNF-*α* agents (*P* value from chi-square test = 0.0547). The mean (±SD) time to discontinuation in all patients whose biologic therapy was interrupted was 17.1 (±17.0) months. The mean (±SD) time to discontinuation was 10.0 (±9.0) months in subjects receiving ADA, 22.5 (±20.4) months for ETA, and 16.3 (±15.5) months for INF (*P* value = 0.0149). Comparisons between biologic therapies revealed statistically significant differences only between ADA and ETA (*P* value = 0.0052) while between ETA and INF (*P* value = 0.0797) and between ADA and INF (*P* value = 0. 3633) the differences were not significant.

The mean (±SD) duration of anti-TNF-*α* treatment for patients whose therapy was not interrupted was 29.0 (±24.4) months. Mean (±SD) persistence of biologic therapy was 16.5 (±15.4) months for ADA, 30.2 (±24.7) months for ETA, and 43.7 (±25.6) months for INF (*P* value < 0.0001). In this case, comparing the biologic therapies revealed statistically significant differences between all pairs of anti-TNF-*α* blockers, between ADA and ETA (*P* value = 0.0002), between ETA and INF (*P* value = 0.0065), and also between ADA and INF (*P* value < 0.0001). Analysis of differences in the duration of anti-TNF-*α* therapy between PsA and AS revealed statistically significant differences between AS and PsA (*P* value = 0.0073) and between Ax-SpA and PsA (*P* value = 0.0467): in general, AS patients were shown to remain on therapy longer than the PsA patients and Ax-SpA patients were shown to remain on therapy longer than PsA patients.

The mean (±SD) duration of anti-TNF-*α* therapy was 20.1 (±20.1) months in women, while in men it was 31.2 (±24.5) months. Only in the group of persistent courses of therapy ANOVA model results did show that gender is an influential factor in duration of therapy (*P* value = 0.0002).

Of the 81 patients who discontinued treatment, 72 patients received at least one other anti-TNF-*α* therapy. Among these, 58 of 72 patients (81.9%) did not need to suspend therapy and forty-five (84.7%) have continued to be responsive to the second anti-TNF-*α* agent for at least six months.

More than 25% of patients were treated concomitantly with DMARDs; however, DMARDs effect on overall persistence of anti-TNF-*α* therapy was not evident: there was no difference in the mean duration of therapy as a function of concomitant therapy (23.5 ± 22.5 months with anti-TNF-*α* therapy plus DMARDs and 26.2 ± 23.4 months with anti-TNF-*α* therapy alone; *P* value = 0.8022). However, analysis of the distribution of patients receiving concomitant DMARDs therapy in terms of persistent versus discontinued courses of therapy revealed that more than twice as many patients were receiving cotherapy during courses that were not interrupted (61/255 (23.9%) for persistent course versus 9/98 (9.2%) for courses that were interrupted; *P* value = 0.0019). In PsA and Ax-SpA there was a statistically significant difference in the group of patients that suspended treatment with regard to concomitant DMARDs (*P* value = 0.030); this significance was not found in comparison to AS and PsA. Data on the mean duration of treatment courses according to concomitant DMARDs status are presented in Table 2.

## 4. Discussion

The results of the present retrospective-observational study, carried out in a routine clinical setting on 353 anti-TNF-*α* treatment courses, show that treatment persistence was favorable both in AS and in PsA; since, after a mean follow-up of 33.7 months, 69.8% of patients (187/268) were still receiving their initial therapy. Our results are in accordance with data from the Spanish registry BIOBADASER and the Italian MonitorNet database [44, 45], which, along with two other studies [46, 47], show a better anti-TNF-*α* retention rate in SpA as compared to rheumatoid arthritis (RA) in a larger number of patients.

In our study, we found that the majority of biologic treatment discontinuation decisions were due to absence of clinical remission 52/98 (53.1%) or occurrence of an adverse event 39/98 (39.8%). Similarly, our results are in line with the data found by Saad et al. in a cohort of 566 patients with PsA, a persistence rate of 75% after 1 year, with the major reasons for discontinuation being inefficacy and adverse events [48].

The results of our study showed that at six-month follow-up, 84.7% of patients who switched to another anti-TNF-*α* agent due to the loss of efficacy over time or due to the occurrence of an adverse event were responsive to the second treatment, in agreement with the current available literature [26–32].

Further, our results showed that axial or peripheral involvement did not seem to be a determinant factor in influencing the anti-TNF-*α* agent retention rate but only the duration of treatment. In particular, in the subset of patients with prevalent axial involvement (AS versus PsA and Ax-SpA versus PsA) we observed a longer duration of biologic therapy. This may be related to the greater influence of TNF-*α* in the pathogenesis of the disease or the earlier introduction of biologic therapy in axial involvement as compared to peripheral arthritis.

In addition, we found that treatment courses were significantly shorter in women than men. This is in keeping with the results of other authors who found a trend towards higher discontinuation rates in women [45, 48].

With regard to concomitant DMARDs use, we found that these drugs were introduced in our cohort approximately twice as frequently among the patients who did not suspend biologic therapy. However, overall we did not observe a significant difference in the duration of anti-TNF-*α* therapy depending on whether a patient was receiving concomitant therapy or not. This could be due to the timing of implementation of DMARDs therapy. The use of cotherapies seemed to be favorably associated with a better persistence rate. The presence of concomitant DMARDs therapies did not differ among disease-type groups, and the suspended treatment group displayed a statistically significant difference in terms of axial involvement (*P* value = 0.030). Two studies have also reported concomitant DMARDs therapy as positive predictors of response to anti-TNF-*α* therapy both in SpA [48] and in RA patients [49].

In conclusion, our data primarily suggest that anti-TNF-*α* agents in PsA and AS cohorts are highly effective and can be associated with a high drug persistence rate.

Furthermore, data from the present study indicate the need to consider the positive effects of switching to another TNF-*α* blocker after discontinuation of an initial anti-TNF-*α* due to the loss of efficacy over time or due to the occurrence of adverse events.

Nevertheless, further studies are needed to determine whether relevant differences exist among the anti-TNF therapies in treatment of spondyloarthritis.

## Figures and Tables

**Table 1:** Demographic and disease characteristics by initial anti-TNF-*α* treatment and for the cohort.

Characteristic	Cohort (*n* = 268)	Adalimumab (*n* = 64)	Etanercept (*n* = 135)	Infliximab (*n* = 69)	*P* value
Patients, *n*					
Female (%)	**126 (47.0)**	39 (60.9)	65 (48.1)	22 (31.9)	0.0034^a^
Median Age, yrs (IQR)	**52 (44–61.5)**	52 (41–59.5)	54 (44–64)	49 (45–57)	0.0366^b^
Mean follow-up, mo (SD)	**33.7 (25.7)**	20.5 (17.4)	32.9 (25.9)	47.2 (25.5)	<0.0001^b^
Disease duration, mean (SD), mo	**98.4 (86.4)**	81.6 (84.1)	95.6 (85.7)	119.7 (86.8)	0.0050^b^
Disease					<0.0001^a^
AS, *n* (%)	**55 (20.5)**	6 (9.4)	20 (14.8)	29 (42.0)	
PsA, *n* (%)	**213 (79.5)**	58 (90.6)	115 (85.2)	40 (58.0)	
Axial involvement					<0.0001^a^
Ax-SpA, *n* (%)	**78 (29.1)**	11 (17.2)	32 (23.7)	35 (50.7)	
PsA, *n* (%)	**190 (70.9)**	53 (82.8)	103 (76.3)	34 (49.3)	
Concomitant therapy					
Any, *n* (%)	**69 (25.7)**	16 (25)	28 (20.7)	25 (36.2)	0.0562^a^
MTX, *n* (%)	**44 (16.4)**	10 (15.6)	17 (12.6)	17 (24.6)	0.0878^a^
LFL, *n* (%)	**13 (4.9)**	2 (3.1)	5 (3.7)	6 (8.7)	0.2223^a^
Other, *n* (%)	**13 (4.9)**	4 (6.3)	6 (4.4)	3 (4.3)	0.8363^a^

SD: standard deviation; IQR: interquartile range; AS: ankylosing spondylitis; PsA: psoriatic arthritis; Ax-SpA; Axial spondyloarthritis; MTX: methotrexate; LFL: leflunomide.

^a^ *P* value from chi-square test and ^b^ *P* value from Kruskal-Wallis test.

**Table 2:** Mean duration of anti-TNF-*α* therapy treatment courses and presence or absence of concomitant DMARDs therapy among patients remaining on therapy and those requiring a therapy change.

		Therapy courses discontinued			Therapy courses ongoing		Total		
	*n* (%)	Mean duration, months (SD)	Concomitant DMARDs, *n* (%)	*n* (%)	Mean duration, months (SD)	Concomitant DMARDs, *n* (%)	*n* (%)	Mean duration, months (SD)	Concomitant DMARDs, *n* (%)
*Anti-TNF-*α* therapy *									
ADA	28 (28.6)	10.0 (9.0)	4 (44.4)	69 (27.1)	16.5 (15.4)	16 (26.2)	97 (27.5)	14.6 (14.2)	20 (28.6)
ETA	41 (41.8)	22.5 (20.4)	3 (33.3)	139 (54.5)	30.2 (24.7)	24 (39.3)	180 (51.0)	28.5 (23.9)	27 (38.6)
INF	29 (29.6)	16.3 (15.5)	2 (22.2)	47 (18.4)	43.7 (25.6)	21 (34.4)	76 (21.5)	33.3 (25.9)	23 (32.8)
*P* value		*0.0149* ^b^	*0.5414* ^c^		<*0.0001* ^b^	*0.0007* ^ c^	*0.0062* ^ a^	<*0.0001* ^b^	*0.0194* ^ c^
*Disease *									
AS	14 (14.3)	21.21 (22.2)	1 (11.1)	54 (21.2)	35.9 (24.8)	15 (24.6)	68 (19.3)	32.9 (24.9)	16 (22.9)
PsA	84 (85.7)	16.5 (16.1)	8 (88.9)	201 (78.8)	27.1 (24.0)	46 (75.4)	285 (80.7)	24.0 (22.5)	54 (77.1)
*P* value		*0.4037* ^b^	*0.7752* ^c^		*0.0976* ^b^	*0.4544* ^c^		*0.0073* ^b^	*0.3945* ^c^
*Axial involvement *									
Ax-SpA	25 (25.5)	18.1 (19.5)	5 (55.6)	76 (29.8)	31.5 (23.7)	18 (29.5)	101 (28.6)	28.2 (23.4)	23 (32.8)
PsA	73 (74.5)	16.8 (16.2)	4 (44.4)	179 (70.2)	27.9 (24.7)	43 (70.5)	252 (71.4)	24.7 (23.1)	47 (67.2)
*P* value		*0.9023* ^ b^	*0.0300* ^c^		*0.1937* ^b^	*0.9538* ^c^		*0.0467* ^b^	*0.3801* ^c^

Total	98 (27.8)	17.1 (17.0)	9 (9.2)	255 (72.2)	29.0 (24.4)	61 (23.9)	353	25.7 (23.2)	70 (19.8)

SD: standard deviation; ADA: adalimumab; ETA: etanercept; INF: infliximab; AS: ankylosing spondylitis; PsA: psoriatic arthritis; Ax-SpA: axial spondyloarthritis; ^a^ *P* values were calculated with the use of a stepwise logistic regression adjusted for gender, concomitant DMARDs, and anti-TNF-*α* therapy. Variables disease and axial involvement were excluded by a stepwise approach (variables being included for *P* < 0.05 and excluded for *P* > 0.1).

^b^ *P* values were calculated by an ANOVA model adjusted for gender, concomitant DMARDs, anti-TNF-*α* therapy, type of disease, and axial involvement.

^c^ *P* value from chi-square test.
